# Defining viral species: making taxonomy useful

**DOI:** 10.1186/1743-422X-11-131

**Published:** 2014-07-23

**Authors:** A Townsend Peterson

**Affiliations:** 1 Biodiversity Institute, University of Kansas, Lawrence, KS 66045, USA

## Abstract

Virus taxonomy at present is best characterized as a categorization of convenience, without a firm basis in the principles of evolutionary biology. Specifically, virus species definitions appear to depend more on tradition and popular opinion among virologists than on firm, quantitative biological evidence. I suggest a series of changes to underlying species concepts that would shift the field from one that simply files viruses away in taxonomic boxes to one that can learn important biological lessons from its taxonomy.

## Introduction

The International Code of Virus Classification and Nomenclature defines a species as “a monophyletic group of viruses whose properties can be distinguished from those of other species by multiple criteria” cix [1]. The Code has undergone several large-scale changes in recent years that improve it and make it more comprehensive and authoritative as a basis for virus taxonomy [1]; the emphasis on monophyly now brings viral species much more in line with those recognized in other major groups (animals, plants). Nonetheless, how the very general definition offered in the Code translates into species-level taxonomies of viruses still has several shortcomings that limit seriously its utility in understanding viral diversity.

### What do we want from a species-level classification?

Most biological classifications aim to achieve three goals: organization, stability, and predictivity. The first two goals are shared with the virus Code: obviously, we require a classification that allows efficient organization of our understanding of virus diversity, and this classification should be as stable and immune to wild change as possible, to avoid introducing confusion in the scientific literature. The third goal, however, is more complex and challenging, and at the same time potentially rewarding: a solid biological classification would offer predictive power about the characteristics of near relatives of known species (see example in [2]).

The Code’s requirement that members of different species taxa be distinguishable by multiple criteria indicates that decisions regarding species status should not based on a single criterion (e.g., considering host association, symptoms caused in host, genetic distance, and other factors). In some sense, this idea makes sense, as it implies that species will not be recognized based on trivial variation in single sets of characters. However, it is also on this point that the Code begins to go awry. That is, one criterion and one criterion alone should indeed dominate: evolutionary independence of evolving lineages [3].

Full summary of viral diversity will also appreciate the potentially diverse ways in which viruses may evolve, which will be obscured by such multiple criteria for species recognition. If a particular lineage differentiates genetically, but retains the same host and causes the same effects in hosts, for example, that combination of diversity and non-diversity is a very interesting feature of that lineage; if such lineages were ignored in viral taxonomy, as would occur under this ‘multiple criterion’ definition in the Code, this phenomenon would be missed and its potential in contributing to viral evolution and innovation would go unappreciated. On the other hand, a single lineage that jumps easily among multiple hosts and causes diverse effects, nonetheless, is not likely to diversify appreciably and may have substantial gene flow among different host populations, and we would have no basis for considering it as multiple species.

My point is that defining species is, by nature, a statement about evolutionary history and how it has structured viral diversity into species taxa. Demanding that species be monophyletic is a first step, but distinguishing species by multiple criteria, if some are nonevolutionary in nature (e.g., host in which occurrence was detected, geographic range, symptomatology), does not aid in appreciating the true phylogenetic diversity of viruses. Other questions fall in realms outside of systematics and taxonomy: e.g., host associations are more questions for a field of viral ecology, and symptomatology is a question for public health or molecular biology of disease. An ideal species-level taxonomy, rather, will reflect evolutionary history, and species limits should be based on evolutionary independence of lineages.

### Examples

Reviewing recent taxonomic summaries of several virus families points to some degree of variation in concepts and protocols. Some virus groups appear to be at such early stages of discovery as to lack a taxonomy, such as the Transfusion Transmitted Virus (TTV) and related viruses, where a recent paper stated that “… a precise and unambiguous naming of the various species listed should be performed” [4], although knowledge of that group appears very scanty. For members of the family *Geminiviridae*, recent taxonomic treatments use a criterion of <89% sequence identity for a virus to be assigned to a separate species [5-7], now with some standardization of methods for calculating sequence identity via decisions about how to treat gap characters [5]; see discussion of sequence identity metrics below. Decisions regarding species status of candidate viruses in these groups, then, appear simply to hinge on levels of sequence divergence in relation to an arbitrary numerical criterion.

An example with considerably more thought and information available is the case of ebola and marburg viruses, family *Filoviridae*, which aptly illustrates the complexities of the situation and the problems that the current system causes, thanks to a detailed recent treatment by the International Committee on Taxonomy of Viruses (ICTV) *Filoviridae* Study Group [8]. The diagnoses of members of each of the species of the family (which in formal taxonomy constitutes the set of qualities that defines membership in the species in question) comprise three pieces of information, none of which, in the end, proves particularly satisfactory as a species diagnosis. First is a statement of where the species is endemic… for example, for *Sudan ebolavirus*, the statement is “being endemic in the Republic of Sudan and the Republic of Uganda.” Note, however, that geographic range is an epiphenomenon of the species, and is not an evolved characteristic of the species—if some traveler were to export this virus unwittingly to, say, Beijing, would the helpful filovirus taxonomist reply, “no, can’t be *Sudan ebolavirus*, because China is not within the diagnosis of the species”? This issue of geographic range being a nonevolved phenomenon of a species is well-known in other sectors of biology, where invasive species are a common example of range expansions that do not necessarily involve evolutionary change [9].

The second characteristic listed for each of the species is number of gene overlaps. Here again, no useful, diagnostic information is provided. The *Reston ebolavirus* genome has two gene overlaps (VP35/VP40, VP24/L), whereas *Taï Forest ebolavirus*, *Sudan ebolavirus*, and *Bundibugyo ebolavirus* are diagnosed as sharing the same two overlaps, plus one more (GP/VP30); however, *Zaire ebolavirus* can show either two or three overlaps [8]. Finally, the genus *Marburgvirus* is stated as having only one gene overlap, but which one is not specified in the formal diagnosis, such that one ends up unsure of the exact characteristics of the genus; at least within *Ebolavirus*, no diagnostic character appears to exist.

Finally, each virus is characterized in terms of sequence differentiation. For example, for species in *Ebolavirus*, the criterion is stated as **≥**30% different from the type sequence of the genus, but <30% different from the type sequence of whichever species is in question. This measure of genetic differentiation is at least a measure of affinity, but the criterion of 30% sequence differentiation (compared with 29% or 31%) is entirely arbitrary, and has no special meaning to the biology, evolution, or any other characteristic of the virus in question.

The end result is curious. Geographic range is a nongenetic epiphenomenon unrelated to the identity of the virus, and no diagnostic differences exist in number or identity of gene overlaps, so sequence differentiation is the only characteristic that more or less diagnoses these virus species. In the end, then, filovirus taxonomy devolves into a single-character diagnosis, *contra* statements in the Code, but a single character that is arbitrary in nature because it does not consider the context of genetic differentiation among viruses within the lineage.

The problem in filoviruses becomes even more acute in *Marburgvirus*. The recent, detailed taxonomic treatment of the family [8] included a section titled, “Marburg virus and Ravn virus are distinct viruses that are members of the same species,” which justifies treating the genus as monospecific. The statementis followed by diagnoses that go as follows: “diverging in genomic nucleotide sequence from the type variant of the type virus of the species *Marburg marburgvirus* (Musoke) by **≤** 10%” *versus* “diverging in genomic nucleotide sequence from the type variant of the type virus of the species *Marburg marburgvirus* (Musoke) by **≥** 10% but different from the type variant of Ravn virus by **≤** 10%.” That is, Ravn virus is ~20% distinct from the type virus of the genus, which is less than 30% but more than 10%, and so this lineage remains unrecognized at the level of species. Why 30% and why 10%? What do these numbers tell us, if anything? Why 10% and not 11%, other than the roundness of the number?

… five lineages of marburgviruses are currently recognized. The genomes of representative marburgvirus variants of one of these lineages differs from all others by up to 21.3% in nucleotide sequence, whereas the genomes of variants from the other four lineages differ from each other only by as much as 0.0–7.8%.

The difference between the ICTV arrangement and the lineage-independence result would probably be only one species (Ravn) in the filoviruses; Peterson and Holder [10] demonstrated lineage independence of Ravn from the remaining known Marburg viruses. Under the ICTV arrangement, the very distinct and lineage-independent Ravn is given the same rank as viruses that have no separate evolutionary history or biological origins, creating a taxonomy that tells us little about the diversity of the virus family. What is lost is a key insight: under the ICTV view, *Marburgvirus* comprises a single species with a broad geographic range, but under the lineage-independence view, *Marburgvirus* comprises two species that are broadly sympatric. These differences have important implications for understanding and anticipating host associations for these viruses [11-13].

### Conclusions: how should the viral code change?

If the goal of taxonomy is to organize biological diversity so as to maximize organization, stability, and predictivity, it is clear that biological criteria must come to dominate the process. We can only hope that viral taxonomy will see the end of Article 3 Rule I-3.3, which states that “… decisions on questions of taxonomy and nomenclature should reflect the majority view of the appropriate virological constituency.” Such decisions should not be a popularity vote, but rather should be based on explicit biological criteria see early example in [14]. I applaud several recent positive steps, though, such as emphasis on monophyly, and linking unique ‘type genomic sequences’ with approved names of individual virus species [15].

Viral taxonomy can change positively by, simply put, bringing in basic principles of evolutionary biology. Virologists have made considerable progress in distinguishing ‘viruses’ (i.e., individual isolates or strains), but higher-level taxa (such as species) can say much of importance about viral diversity. That is, a species-level taxonomy should be viewed as a statement of the diversity that has evolved over the history of a group. Two immediate corollaries are evident, both as regards decisions about species status.

First, a frequent statement against recognition of a virus species is that it still shows antigenic cross-reactivity with other (recognized) lineages (similar statements are made about host associations or symptomatology, although these characteristics are not necessarily evolved features of the virus). The fallacy of this argument was pointed out decades ago in animal systematics, as regards the relevancy of reproductive compatibility of animal species in setting species limits [3]: antigenic cross-reactivity is an ancestral (plesiomorphous) trait of the broader lineage that gave rise to the two forms in question. The fact that they have not yet evolved antigenic non-cross-reactivity does not say that they have not evolved significantly and independently in other regards, to the point that they merit species status. Rather, use of such plesiomorphic traits in recognizing species (or not) is equivalent to lumping all rodents into a single species because they all have hair and mammary glands. A more positive approach is to assume that the two candidate forms are the same species until proof emerges of independent evolution in the two lineages, such as fixing derived (apomorphic) traits in each lineage—these traits could include fixing sufficient numbers of bases in a gene sequence, or any other evolved, derived feature of the virus.

Second, species limits can and should be established quantitatively based on estimates of lineage independence, which in essence depend on measures of genetic diversity between lineages relative to genetic diversity within lineages. That is, a criterion of 30% sequence differentiation means little if within-lineage diversity reaches 29.9%. Molecular systematists working with vertebrates have developed a series of approaches to determining lineage independence [16-20] that take into account variation and differentiation both within and between lineages, although the specifics of these criteria should depend on the specific evolutionary mechanisms that dominate in a particular group [3]; these approaches can be adapted to evolutionary mechanisms of viruses [indeed, ref [10] prototyped such an analysis of the Marburg-Ravn situation, and found that the two lineages are clearly evolving independently]. A first step is to use appropriate evolutionary model-based measures of evolutionary divergence, as the simple sequence difference currently being employed do not take into account multiple substitutions [21]: what was a bad idea for vertebrates [22], where interspecific divergences are well below 10%, is a really bad idea for viruses, where divergences are commonly much deeper, with much more opportunity for multiple substitutions. A much better idea would be to appeal to coalescent approaches that can consider historical and present population sizes in deriving much more meaningful measures of evolutionary divergence [23]. This general focus on virus species as evolving lineages would be a healthy change for the field of viral systematics.

A final point is that this commentary is a view from the outside, and I am sure that it will be received as such by many in the community: an unwanted intrusion into the business of others. Indeed, a number of virus-specific points should be considered carefully: e.g., (1) very rapid and often massively varying rates of evolution and the confusions that can result, (2) large-scale propensity of viruses for reassortment or recombination, and (3) the large number of viruses for which molecular sequence data are not yet available (which should be designated as *incertae sedis*). Nonetheless, more generally, it is clear that virus taxonomy is failing in a number of regards. Viewing virus diversity as the present-day snapshot of a wide array of evolving lineages would not change the picture drastically, but would change the picture of current virus diversity in ways that may not be expected. Most importantly, definitions of species-level entities in the virus world would be based on evolutionary concepts, rather than a mixture of evolutionary and nonevolutionary concepts and criteria.
